# A Novel Approach to Unraveling the Apoptotic Potential of Rutin (Bioflavonoid) via Targeting *Jab1* in Cervical Cancer Cells

**DOI:** 10.3390/molecules26185529

**Published:** 2021-09-12

**Authors:** Pratibha Pandey, Fahad Khan, Faisal Abdulrahman Alzahrani, Huda A. Qari, Mohammad Oves

**Affiliations:** 1 Department of Biotechnology, Noida Institute of Engineering and Technology, Greater Noida 201306, India; pratibhapandey.bio@niet.co.in or; 2 Department of Biochemistry, Faculty of Science, Embryonic Stem Cells Unit, King Abdulaziz University, Jeddah 21589, Saudi Arabia; faahalzahrani@kau.edu.sa; 3 Department of biological Science, Faculty of Sciences, King Abdulaziz University, Jeddah 21589, Saudi Arabia; hagri@kau.edu.sa; 4 Center of Excellence in Environmental Studies, King Abdulaziz University, Jeddah 21589, Saudi Arabia

**Keywords:** cervical cancer, rutin, *Jab1*, apoptosis, *Caspase-3*, ROS, oncogene

## Abstract

Rutin has been well recognized for possessing numerous pharmacological and biological activities in several human cancer cells. This research has addressed the inhibitory potential of rutin against the *Jab1* oncogene in SiHa cancer cells, which is known to inactivate various tumor suppressor proteins including *p53* and *p27*. Further, the inhibitory efficacy of rutin via *Jab1* expression modulation in cervical cancer has not been yet elucidated. Hence, we hypothesized that rutin could exhibit strong inhibitory efficacy against *Jab1* and, thereby, induce significant growth arrest in SiHa cancer cells in a dose-dependent manner. In our study, the cytotoxic efficacy of rutin on the proliferation of a cervical cancer cell line (SiHa) was exhibited using MTT and LDH assays. The correlation between rutin and *Jab1* mRNA expression was assessed by RT-PCR analysis and the associated events (a mechanism) with this downregulation were then explored via performing ROS assay, DAPI analysis, and expression analysis of apoptosis-associated signaling molecules such as *Bax*, *Bcl-2*, and *Caspase-3* and -9 using qRT-PCR analysis. Results exhibit that rutin produces anticancer effects via inducing modulation in the expression of oncogenes as well as tumor suppressor genes. Further apoptosis induction, caspase activation, and ROS generation in rutin-treated SiHa cancer cells explain the cascade of events associated with *Jab1* downregulation in SiHa cancer cells. Additionally, apoptosis induction was further confirmed by the FITC-Annexin V/PI double staining method. Altogether, our research supports the feasibility of developing rutin as one of the potent drug candidates in cervical cancer management via targeting one such crucial oncogene associated with cervical cancer progression.

## 1. Introduction

Cancer of the cervix has been recognized as one of the most frequently detected cancers worldwide, and several studies are in the pipeline for elucidating efficient therapeutic approaches against this malignancy [1,2]. Inhibition of cervical cancer progression with phytochemicals via modulating numerous signaling pathways has been validated by various published studies [3,4]. Phytochemicals (plant-based products) have received wider significance in exploring potent therapeutic approaches against numerous carcinomas with enormous health benefits [5]. Toxicity associated with several chemotherapeutic approaches has raised the need for the elucidation of better anticancer drugs [6]. Several studies have reported the efficacy of natural phytocompounds as an alternative to surgery and chemotherapeutic drugs. Various natural compounds, including carvacrol, rutin, paclitaxel, vincristine, hesperidin, etoposide, and vinblastine, have shown better medicinal benefits and have been utilized clinically [7].

Phytocompounds display their anticancerous potential either by inducing growth arrest or modulation in several signaling pathways in numerous cancerous cells. Amongst several natural or plant-derived compounds, flavonoids have gained wider interest because of their remarkable range of biological benefits including antiallergic, antioxidant, antiangiogenic, antimutagenic, anti-inflammatory, antibacterial, and anticancer activity [8,9,10]. Flavonoids (polyphenolic compounds) have been categorized mainly into flavanones, isoflavones, flavones, flavonols, chalcones, flavanonols, and flavonols. Their ability to induce apoptosis, block the cell cycle, or inhibit angiogenesis makes them strong promising agents in cancer management [11,12]. Rutin (a flavonol) has been abundantly reported in various plants including passionflower, apple, buckwheat, and tea [13,14,15]. Current research has demonstrated its multispectral pharmacological potential in the treatment of numerous diseases such as hypertension, cancer, hypercholesterolemia, and diabetes. Rutin has exhibited various pharmacological activities, viz., cytoprotective, antioxidant, anticarcinogenic, vasoprotective, cardioprotective, and neuroprotective activities [16,17,18,19,20].

Elucidation of potent molecular targets to distress cervical carcinogenesis and its intervention by plant-derived (or dietary) agents is a vital part of chemoprevention. Recent advancements in the treatment approach for cancer management have impelled us to utilize the potential of phytocompounds against a protein that has been responsible for several carcinomas. Thus, our study is focused on *Jab1*, which is a multifunctional protein involved in the progression of numerous cancers. To date, a very limited number of natural compounds (phytocompounds) against *Jab1* in cervical cancer have been reported. Rutin has exhibited significant anticancerous efficacy at a very low dose against several cancer cells including prostate cancer, breast cancer, cervical cancer, and colon cancer. However, the role of rutin against *Jab1* has been unexplored. Therefore, our research is focused on exploring the mechanism behind the mode of action of rutin targeting *Jab1* in cervical cancer.

## 2. Materials and Methods

### 2.1. Experimental Requirements

Fetal bovine serum (FBS), rutin, MTT (5-dimethylthiazol-2-yl)-2, 5-diphenyltetrrazo-lium bromide), DMEM, and DMSO (dimethylsulfoxide) were procured from Sigma-Aldrich (St. Louis, MO, USA). Antibiotics (penicillin and streptomycin), DCFH-DA (2′,7′-dichlorodihydrofluorescein diacetate), DAPI (4′,6-diamidino-2-phenylindole), MitoTracker Red CMX-Ros and Rhodamine123 (Rh123) were procured from Sigma-Aldrich (St. Louis, MO, USA). SiHa cancer cells were cultured in high-glucose DMEM (Gibco, TN, USA) media supplemented with 100 g/mL of antibiotics (streptomycin and penicillin) and 10% fetal bovine serum (FBS) (Gibco, TN, USA). Cultured cells were then incubated in an incubator (at 37 °C and 5.0% CO_2_ atmosphere).

### 2.2. MTT Assay

The inhibitory effect of rutin on SiHa cells was analyzed by MTT assay as reported in previous studies [21]. In brief, SiHa cells were incubated for attachment in an incubator for 24 h (at 37 °C) and then eventually treated with rutin (40–200 µm) for a further 24 h. Each well was then supplemented with 10 μL of MTT (5 mg/mL) dye and incubated at 37 °C for 4 h. Formazans (or purple-colored precipitates) were dissolved in DMSO (100 μL) with gentle shaking. Finally, the optical density absorbance of the reaction mixture was quantified at 490 nm using an ELISA plate reader (Bio-Rad, Hercules, CA, USA) after complete dissolution and cell survival was evaluated as a percentage over the untreated control.

### 2.3. Extracellular Lactate Dehydrogenase (LDH) Activity Analysis in Rutin-Treated SiHa Cells

LDH activity was investigated by using Cytotoxicity Cell Death Kit (as per the manufacturer’s protocol, Sigma, Mundelein, IL, USA). SiHa cells were then exposed to selective rutin doses. Thereafter, supernatant or sample solution was further collected to determine LDH activity in both control and treated SiHa cancer cells. Absorbances of both the treated and untreated samples were then recorded at 490 nm using a microplate reader (Bio-Rad, Hercules, CA, USA).

### 2.4. Investigation of Nuclear Morphology in Rutin-Treated Cells

DAPI (fluorescent nuclear dye) was used for determining the apoptotic potential of rutin on SiHa cancer cells as per the protocol described in our previous studies [22]. SiHa cancer cells after treatment with selective rutin doses (0, 80, 120, and 160 µm) were left to incubate for 24 h. Thereafter, treated SiHa cancer cells were exposed to paraformaldehyde (3.5%) and 0.05% (*v*/*v*) Triton X-100 for permeabilization and fixation. Lastly, morphological changes (fluorescent nuclei) were observed using an inverted fluorescence microscope (Thermo Fisher Scientific, Waltham, MA, USA).

### 2.5. Quantification of Apoptosis by Annexin V-FITC/PI Assay

Rutin-induced apoptosis induction in SiHa cancer cells was quantified using an Annexin V-FITC/PI assay kit (BD Biosciences, Billerica, MA, USA). Briefly, SiHa cancer cells (in 12-well plates) at a density of 5 × 10^5^ cells per mL wer treated with rutin doses (0, 80, 120, and 160 µm) for 48 h. After 48 h of incubation, SiHa cells were washed twice with cold PBS (phosphate-buffered saline) buffer and then the cells were re-suspended in binding buffer (500 μL). Thereafter, cells were stained with annexin V-FITC/PI and left to incubate for 15 min in the dark at room temperature. Lastly, the stained cells were analyzed by using flow cytometry.

### 2.6. Investigation of Caspases Activities in Rutin-Treated SiHa Cells

Caspase activities in rutin-treated SiHa cells were assessed by a caspase colorimetric assay kit (BioVision, Milpitas, CA, USA). Both untreated (control) and rutin-treated SiHa cancer cells were lysed in chilled buffer (cell lysis) and incubated for 10 min on ice. Further, centrifugation was performed at 10,000× *g* for 1 min to obtain supernatant, which was kept on ice for instant assay. Then, 50 μL of lysate was aliquoted into 96-well plates with 50 μL of reaction buffer containing 10 mm DTT. DEVD-pNA (5 μL) substrate was then added into each well and left to incubate for 1 h at 37 °C. Absorbance was recorded at 405 nm on a microtiter plate reader. Percent increase in *Caspase-3* activity was determined by comparing the result of treated cells with untreated cells (level of uninduced control).

### 2.7. Investigation of the Effect of Caspase (Caspase-3 and Caspase-9) Inhibitors

In order to assess the role of caspase activation in rutin-induced apoptosis, SiHa cervical cancer cells were pretreated with 50 μM of both *Caspase-3* inhibitor (Z-DEVD-FMK) and *Caspase-9* inhibitor (Z-LEHD-FMK) for 2 h and then eventually treated with rutin at selective doses for 24 h. Lastly, cell viability was evaluated by using an MTT assay as explained in the MTT section.

### 2.8. Investigation of MMP (Mitochondrial Membrane Potential) in Rutin-Treated SiHa Cells

Two fluorescent dyes including mitochondrial-specific MitoTracker Red and Rhodamine 123 were used to observe MMP variation in rutin-exposed SiHa cells as per the procedure described by Pandey et al., 2020 [23]. Cells were treated with varied rutin doses (80–160 μM and left to incubate for 12 h. Thereafter, SiHa cancer cells were washed (with PBS) and further exposed to paraformaldehyde for 15 min at 37 °C for cell fixation. Lastly, rutin-exposed SiHa cells were stained with both the fluorescent dyes MitoTracker Red (25 ng/mL) and Rhodamine (5 μg/mL) to record MMP variation under fluorescence microscope (EVOS Floid cell imaging station, Thermo Fisher Scientific, Waltham, MA, USA).

### 2.9. Measurement of Cytochrome C Level

ELISA (solid-phase enzyme-linked immunosorbent) assay was used to measure cytosolic cytochrome c level in rutin-treated SiHa cells. The assay was carried out as per the instructions provided in the Cytochrome C Human ELISA Kit (Invitrogen, Waltham, MA, USA). In brief, SiHa cancer cells (1 × 10^7^ cells per mL) were treated with selective rutin doses and left to incubate for 48 h. After cell lysis, the supernatant was assayed by measuring the optical density of samples at 450 nm using an ELISA plate reader (BioTek, H1M, Winooski, VT, USA). The standard curve was plotted by considering the absorbance values of dilutions of a cytochrome c standard.

### 2.10. Measurement of Intracellular ROS (Reactive Oxygen Species) Level

The DCFH-DA (dye) method was used for the evaluation of ROS generation in rutin-treated SiHa cancer cells [23]. SiHa cancer cells (1.5 × 10^4^) were then incubated at 37 °C for 24 h. After exposure of SiHa cells to rutin (80–160 µm) for 12 h, DCFH-DA 10 (µm) was then added to the rutin-treated SiHa cells and incubated at 37 °C for 30 min. Images of rutin-exposed cells were recorded after washing off excessive dye, using an inverted fluorescence microscope (Japan). Quantitative estimation of intracellular ROS generation was performed by incubating rutin-exposed cells with dye (10 µm) at 37 °C for 30 min. A microplate reader (USA) was utilized to record the fluorescence intensity at both emission (528 nm) and excitation wavelength (485 nm). Values were then expressed as percentage of fluorescence intensity relative to control.

### 2.11. Investigation of ROS Inhibitor, NAC (N-Acetyl-L-Cysteine) Efficacy

NAC was used to support the intracellular ROS generation in rutin-treated SiHa cervical cancer cells. Firstly, SiHa cancer cells were pretreated with NAC (10 mm) for the duration of 2 h followed by exposure to rutin for 12 h. Treated cells were then washed with PBS and stained with dye DCFH-DA (10 mm). Treated cells were then left to incubate at 37 °C for 30 min. A microplate reader was used to record the fluorescence intensity at both emission (528 nm) and excitation (485 nm) wavelengths. However, to further explore the correlation of ROS generation with apoptosis in rutin-treated SiHa cancer cells, we elucidated rutin efficacy in SiHa cells in the presence of 10 mm of *N*-acetyl-*L*-cysteine via the MTT assay.

### 2.12. Real-Time PCR Analysis

In order to elucidate whether rutin treatment displayed any modulatory effect on transcription of pro-apoptotic and anti-apoptotic genes in SiHa cells, mRNA expression of all selected target genes in both control (DMSO) or rutin-exposed cells was investigated. Seeded SiHa cells were then treated with rutin, left to incubate for 24 h, and thereafter trypsinized. The obtained cell suspension was then centrifuged twice, and the cell pellet was stored at −80 °C for further analysis. After 24 h post treatment with rutin, total RNA was extracted using TRIzol Reagent (according to the protocol of Invitrogen). Super Script III one-step RT-PCR kit (Invitrogen) with Platinum Taq DNA polymerase was then used to investigate the relative expression of both treated and control samples and evaluated by 2(^ΔΔCt^) method. Selected primers utilized in this research study are shown in Table 1.

### 2.13. Statistical Analyses

Data analysis was performed using Prism software (GraphPad Prism version 7.0, GraphPad Software, Inc., San Diego, CA, USA). Statistical analyses were performed using one-way ANOVA. Error bars for SEM are shown. Where indicated in the figures, degrees of *p*-value significance are as follows: * *p* < 0.01 and ** *p* < 0.001.

## 3. Results

### 3.1. Rutin-Induced Antiproliferative Activity in SiHa Cervical Cancer Cells

**Figure 1:**
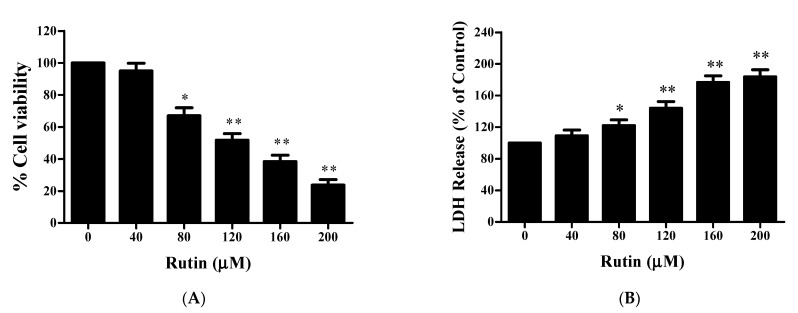
Effect of rutin on the viability of cervical cancer SiHa cells. (**A**) Bar diagram presenting cell viability of SiHa cells exposed to various concentrations of rutin (0–200 μM) for 24 h as assessed by MTT assay. (**B**) Cytotoxic effects of rutin analyzed by lactate dehydrogenase (LDH) enzymatic activity in human cervical cancer SiHa cells. The results are represented as mean ± SEM of three independent experiments (* *p* < 0.01, ** *p* < 0.001 compared with control group).

The MTT assay was utilized to assess the antiproliferative efficacy of rutin on the proliferation of SiHa cells. Figure 1 exhibits a significant reduction in cell viability of rutin-treated SiHa cells in a dose-dependent manner in comparison to control (Figure 1A). Origin software was further used to evaluate the IC_50_ value (IC_50_ = 125.43 μM) for screening respective rutin doses for further experimental procedures. The results showed that rutin treatment at 80, 120, 160, and 200 μM significantly reduced the cell viability of SiHa cells to around 67.05% ± 5.03%, 51.76% ± 4.15%, 38.36% ± 3.98%, and 23.69% ± 3.45%, respectively, as compared to control. Additionally, an LDH activity assay was employed to confirm cell cytotoxicity by estimating LDH release in the disrupted cell membrane. Figure 1B revealed a significant increase in dead cells after 24 h of rutin treatment. Altogether, both cytotoxicity and cell proliferation assays strongly supported the anticancer potential of rutin against SiHa cervical cancer cells.

### 3.2. Rutin Inhibits the Growth of SiHa Cells via Downregulation of Jab1 and Upregulation of p27

**Figure 2:**
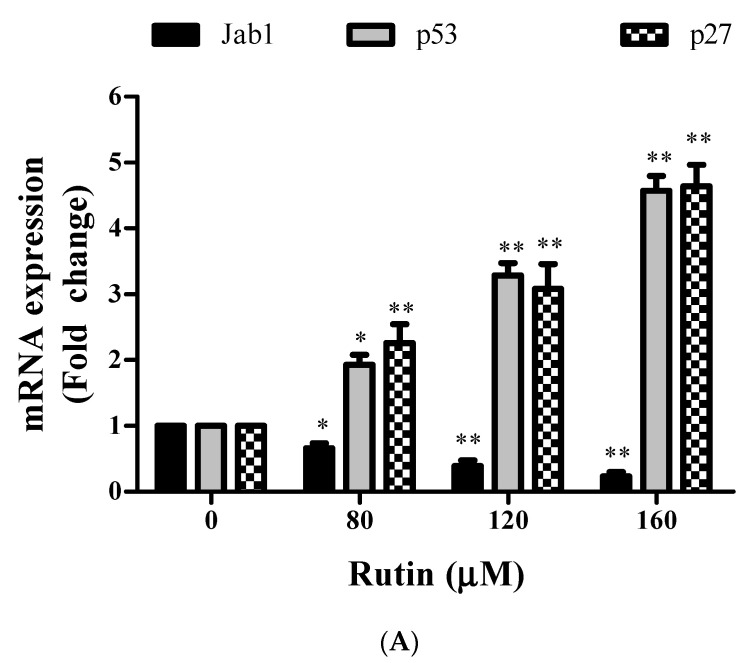

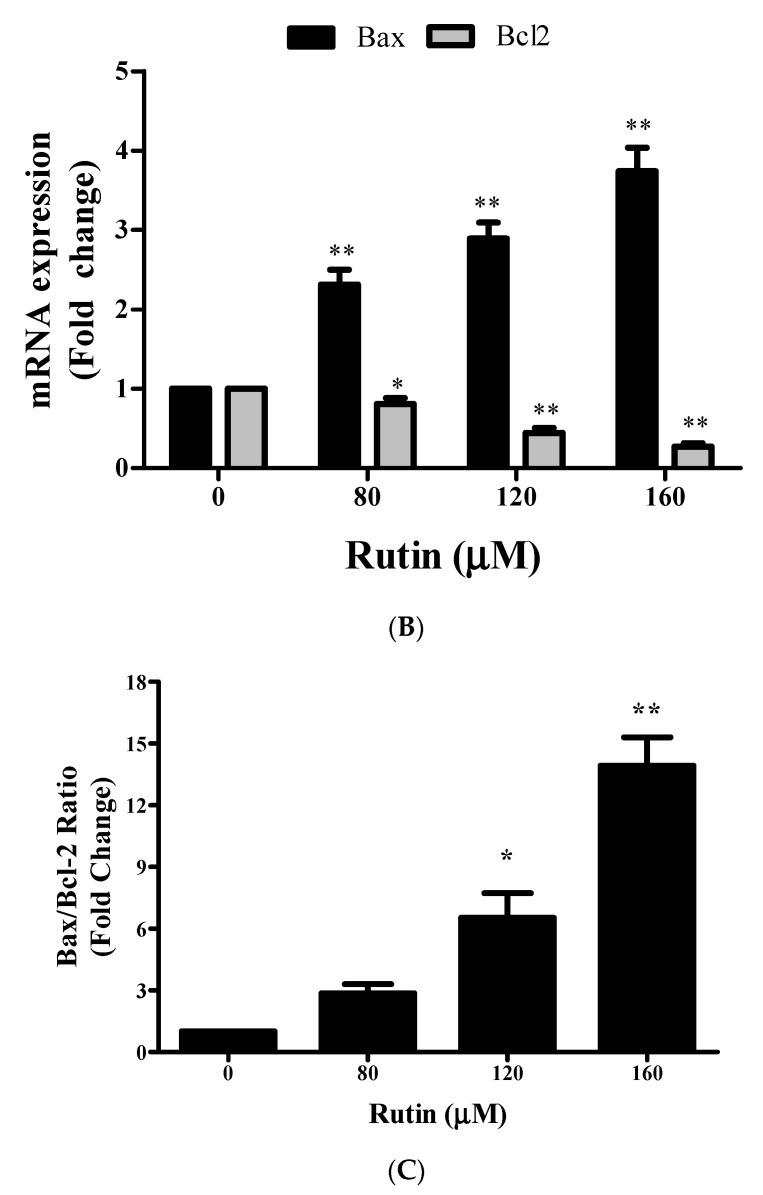
Effect of rutin on gene expression of mRNA expression of target genes. (**A**) The mRNA expression of *Jab1*, *p27*, and *p53* in rutin-treated SiHa cells (80, 120, and 160 µM) was investigated by qRT PCR. (**B**) The mRNA expression of proapoptotic gene *Bax*, antiapoptotic gene Bcl2, and (**C**) *Bax*/Bcl2 ratio in cervical cancer cells treated with rutin (80, 120, and 160 µM) was investigated by qRT PCR. The results are represented as mean ± SEM of three independent experiments (* *p* < 0.01, ** *p* < 0.001 compared with control group).

RT-PCR is used to elucidate the effect of rutin treatment on *Jab1* mRNA transcripts levels that have been crucially associated with cervical cancer progression. *Jab1* expression was determined after 24 h of rutin treatment in SiHa cells. *Jab1* has also been associated with the downregulation of tumor suppressor genes. This has further projected us to investigate the effect of *Jab1* mRNA downregulation on *p27*. Figure 2 depicts that rutin treatment resulted in a significant reduction in *Jab1* expression levels in cervical cancer SiHa cells. Eventually, significant *p27* mRNA upregulation was also observed in rutin-treated SiHa cells in a dose-dependent manner.

### 3.3. Rutin Induces Apoptosis in SiHa Cancer Cells through Upregulation of p53, Bax, and Downregulation of Bcl-2

Real-time PCR was employed to analyze the mRNA expression of *Bcl-2*, *Bax*, and *p53* in rutin-treated cervical cancer cells. RT-PCR analysis exhibited significant upregulation in the mRNA expression level of *p53* and *Bax* in a dose-responsive manner in rutin-treated SiHa cells (Figure 2A,B). Rutin treatment also resulted in considerable downregulation in the mRNA expression level of *Bcl-2* (anti-apoptotic gene) in SiHa cells in a dose-responsive manner (Figure 2C). These findings indicate that upregulation of *p53* and *Bax* genes and downregulation of anti-apoptotic *Bcl-2* genes are involved in the mechanism behind the rutin-mediated induction of apoptosis in cervical cancer cells.

### 3.4. Mitochondrial-Mediated Apoptosis Induction in Rutin-Treated SiHa Cancer Cells

**Figure 3:**
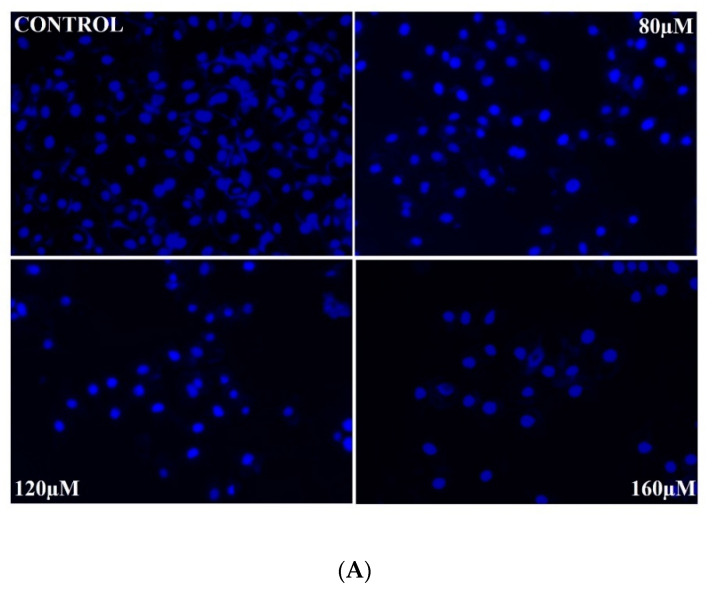

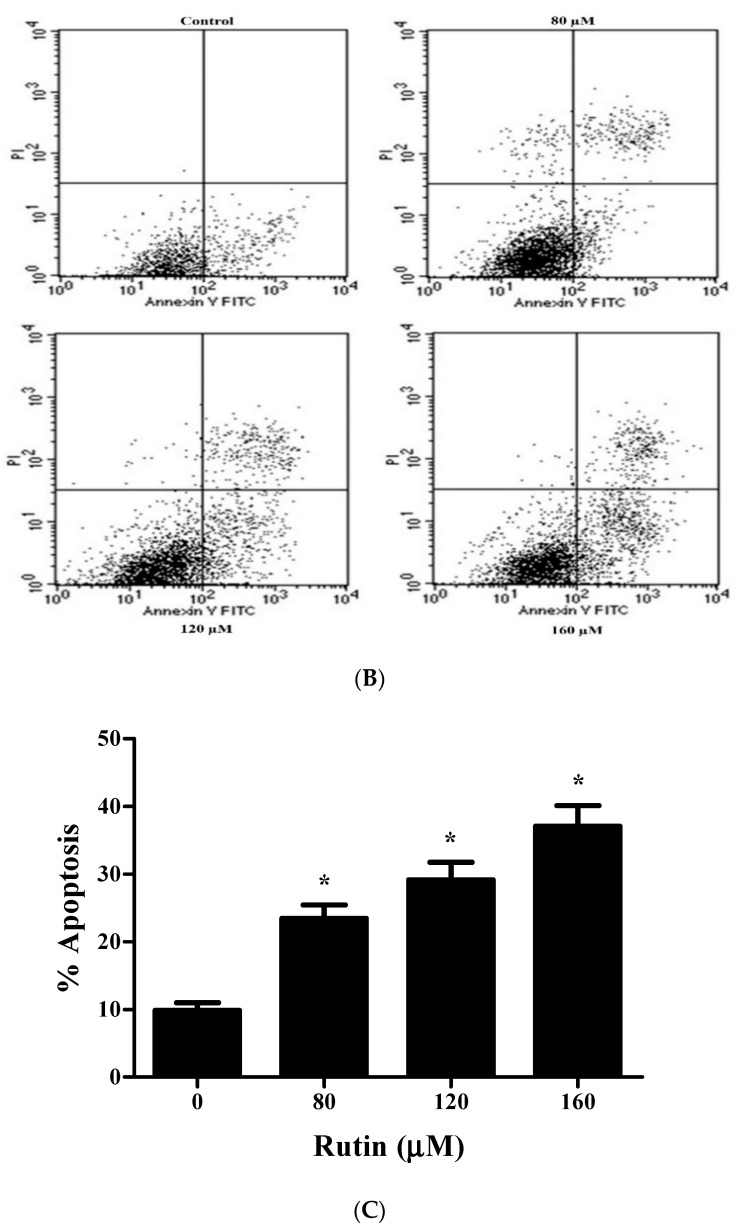
Apoptosis induction in rutin-treated SiHa cancer cells. (**A**) Nuclear morphology alteration of rutin-treated SiHa cells analyzed by DAPI staining method and visualized under fluorescence microscope. (**B**) Histogram representing percent apoptosis in rutin-treated SiHa cells for 24 h examined by flow cytometric analysis. (**C**) Percentage of rutin-induced apoptotic cells in cervical cancer SiHa cells examined by Annexin V-FITC/PI assay (* *p* < 0.05 compared with control group).

DAPI staining was performed to elucidate the preliminary events of apoptosis associated with nuclear morphology in rutin-treated SiHa cells. Apoptosis was observed after 24 h of rutin treatment in SiHa cancer cells using DAPI dye. Apoptotic bodies were characterized by fragmented and condensed nuclei. Rutin-treated cells showed apoptotic bodies, while untreated or control cells showed no significant apoptosis (Figure 3A). Annexin V-FITC/PI staining was performed to verify the apoptotic rate of rutin in SiHa cells by flow cytometry, and the level of early and late apoptotic cells were quantified. Figure 3B,C exhibits the increased apoptotic rate in rutin-treated SiHa cells (23.48%, 29.15%, and 37.09%) in comparison to that in control cells (9.87%) after 24 h of treatment with 80, 120, and 160 µm of rutin, respectively.

**Figure 4:**
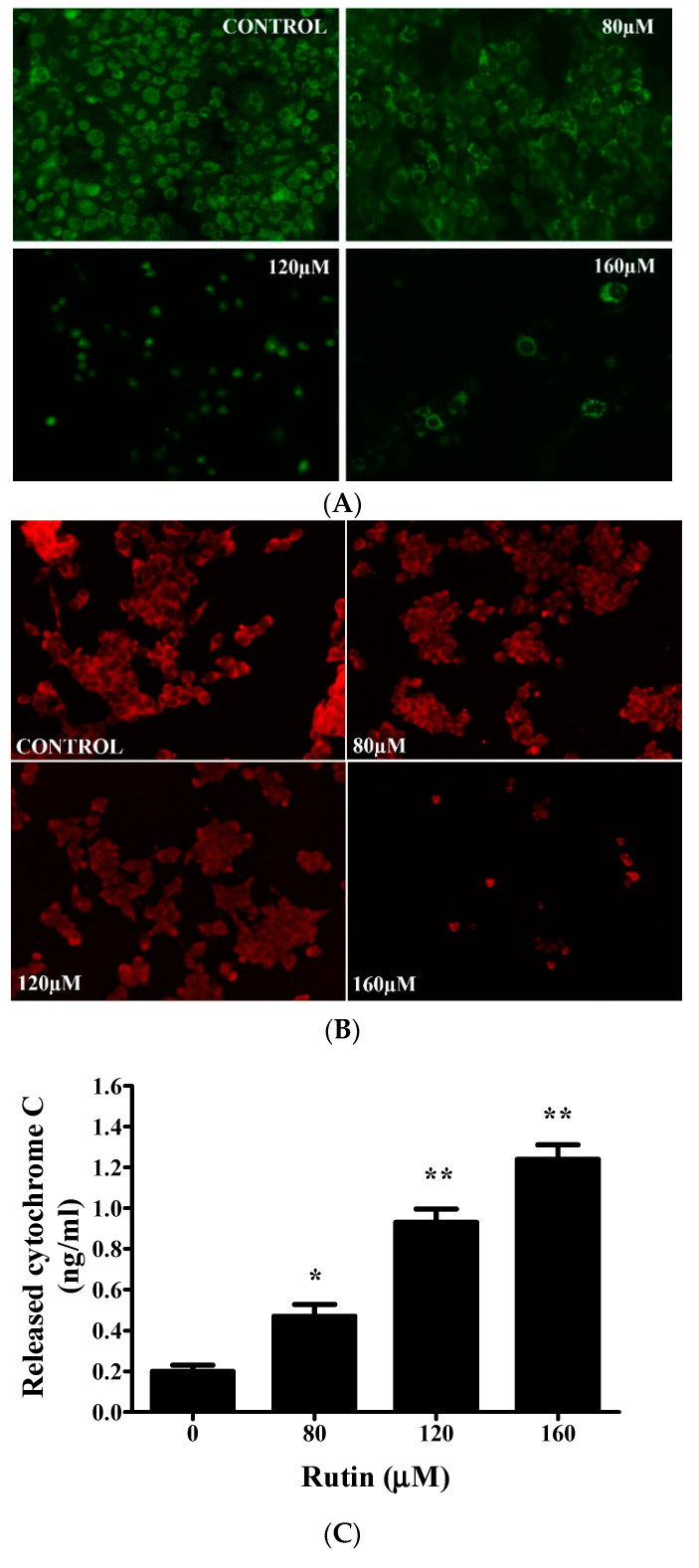
Rutin significantly induces mitochondrial-mediated apoptosis in cervical cancer cells. (**A**) Rutin-induced mitochondrial membrane potential (MMP) disruption in cervical cancer SiHa cells analyzed by staining with fluorescent dye Rh123 and (**B**) MitoTracker Red. (**C**) Rutin induces the release of cytochrome c in cervical cancer SiHa cells assessed by a human cytochrome c enzyme-linked immunosorbent assay kit. The results are represented as mean ± SEM of three independent experiments (* *p* < 0.01, ** *p* < 0.001 compared with control group).

Mitochondrial membrane depolarization (Δψm) potential was analyzed in both untreated and rutin-treated SiHa cells with MitoTracker Red stain. Figure 4A, B strongly presented the fact that rutin treatment resulted in a significant reduction (in a dose-dependent manner) in Δψ in SiHa cells, which was represented by a decrease in red fluorescence intensity. As rutin demonstrated stimulated nuclear condensation and mitochondrial membrane potential disruption, the present study further investigated the involvement of mitochondria in apoptosis induction via the release of cytochrome c in rutin-treated SiHa cells. Figure 4C displays an enhanced cytosolic cytochrome c level in rutin-treated SiHa cells in comparison to that in untreated control cells. These results further suggested that mitochondrial-mediated apoptosis induction could be one of the possible mechanisms behind the growth inhibitory effect of rutin in cervical cancer SiHa cells.

### 3.5. Caspase-Mediated Apoptosis Induction in Rutin-Treated SiHa Cancer Cells

**Figure 5:**
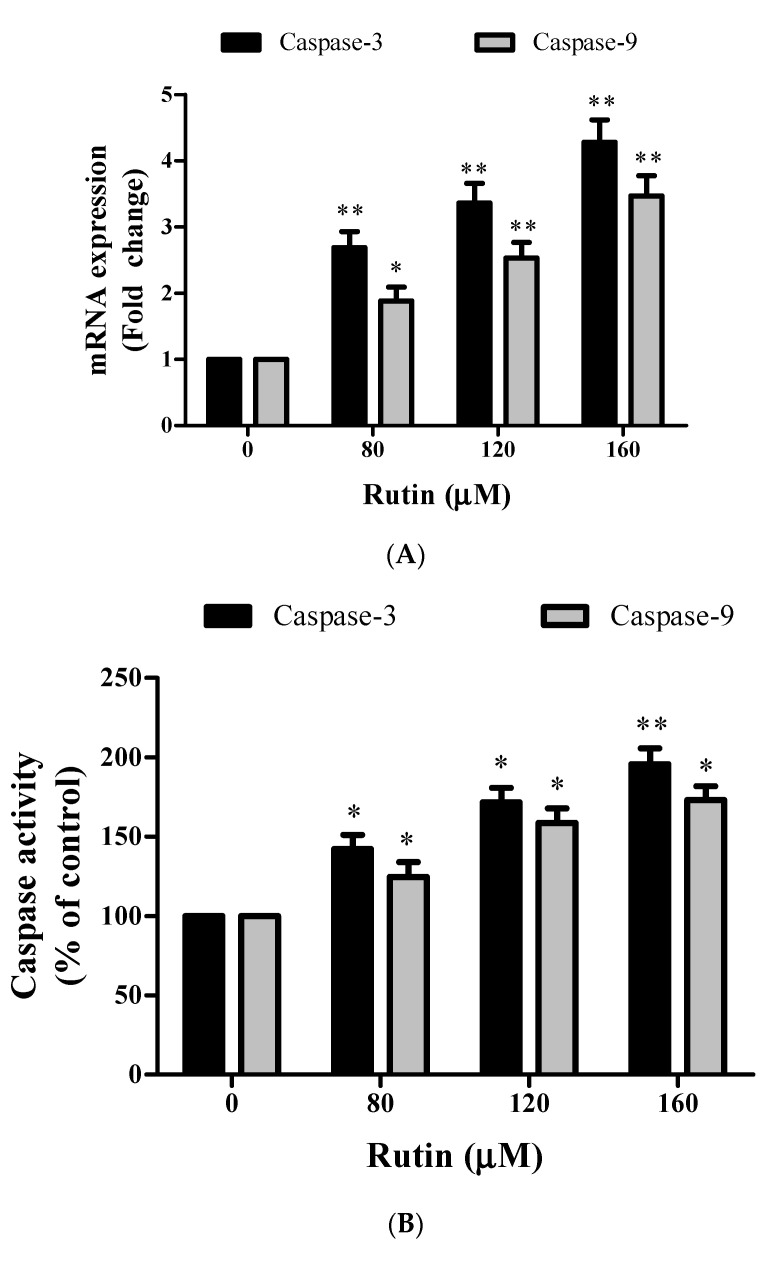

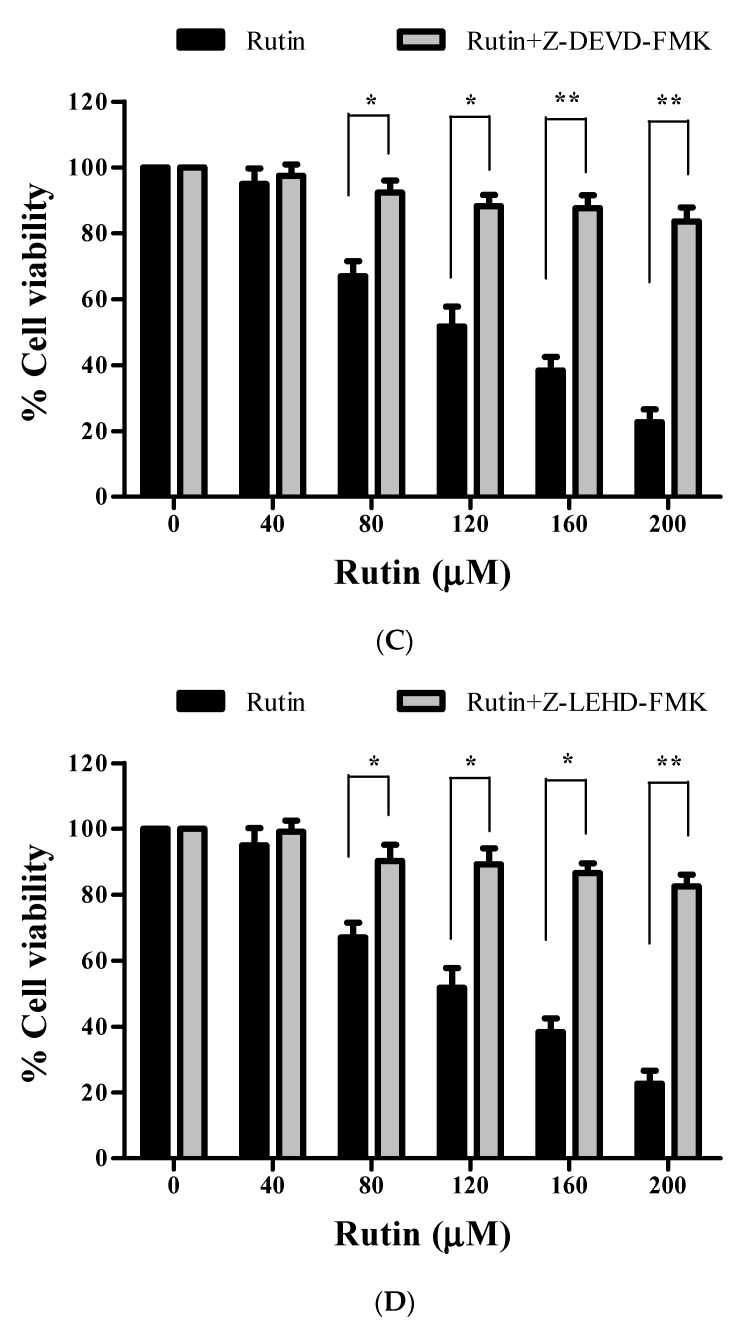
Rutin significantly induces caspase-mediated apoptosis in cervical cancer cells. (**A**) The mRNA expression of *Caspase-3* and -9 in cervical cancer cells treated with rutin (80, 120, and 160 µM) was investigated by qRT PCR. (**B**) Enhanced activity of *Caspase-3* and -9 in rutin-treated SiHa cells examined by caspase colorimetric kits using specific substrates. (**C**,**D**) Percent cell viability of SiHa cells pretreated with respective caspase inhibitors then treated with various concentrations of rutin (40–200 μM) for 24 h assessed by MTT assay. The results are represented as mean ± SEM of three independent experiments (* *p* < 0.01, ** *p* < 0.001 compared with control group).

Caspases are a family of cysteine proteases that triggers apoptosis via cleaving proteins at aspartic acid residues. To further investigate the underlying mechanism of apoptosis induction, we further investigated the involvement of caspases in apoptosis induction in SiHa cells, real-time qPCR and colorimetric analysis were performed. These results indicated that rutin treatment significantly stimulated the *Caspase-3* and -9 activities after 24 h as observed by mRNA expression and percent activation (Figure 5A,B).

To illustrate whether rutin-induced cytotoxicity in SiHa cancer cells was associated with the activation of caspases (*Caspase-3* and -9), SiHa cells were pretreated with 50 µm of *Caspase-3* and -9 inhibitors (Z-DEVD-FMK and Z-LEHD-FMK) for 2 h and then treated with selective doses of rutin for 24 h (Figure 5C,D). The MTT assay was used to assess the cell viability as described above. Pretreatment with both the caspase inhibitors potentially reduced the cytotoxicity in SiHa cancer cells caused by rutin treatment. These results have supported the crucial role of caspase activation in rutin-induced apoptosis in cervical cancer cells via a caspase-dependent pathway.

### 3.6. Effect of Rutin on ROS Generation in Apoptosis Induction

**Figure 6:**
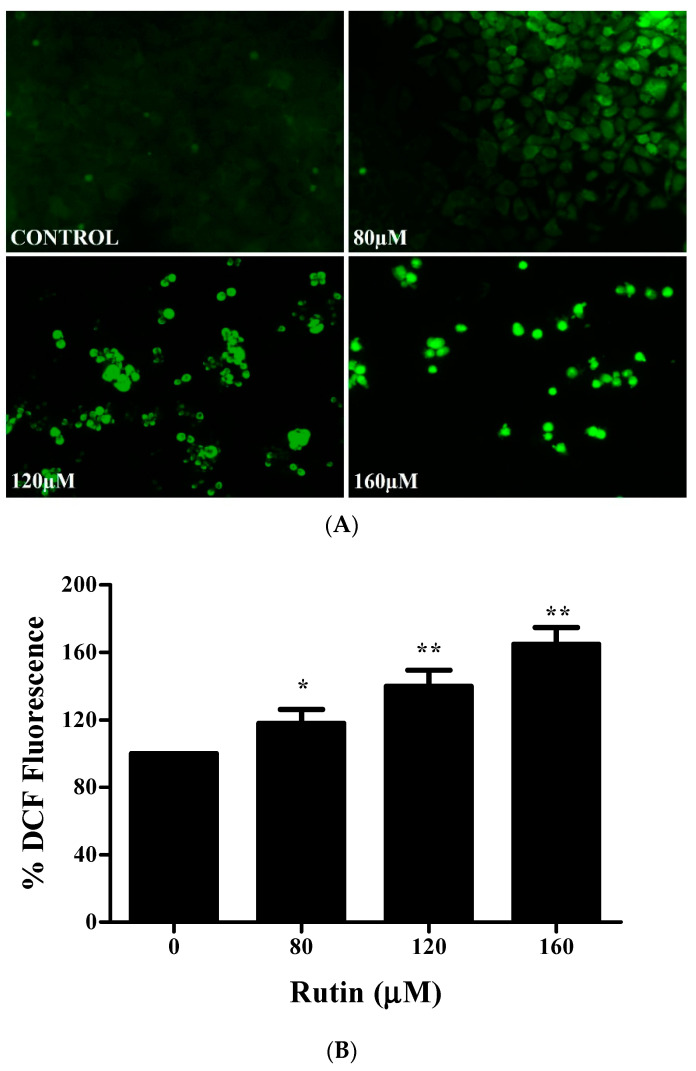

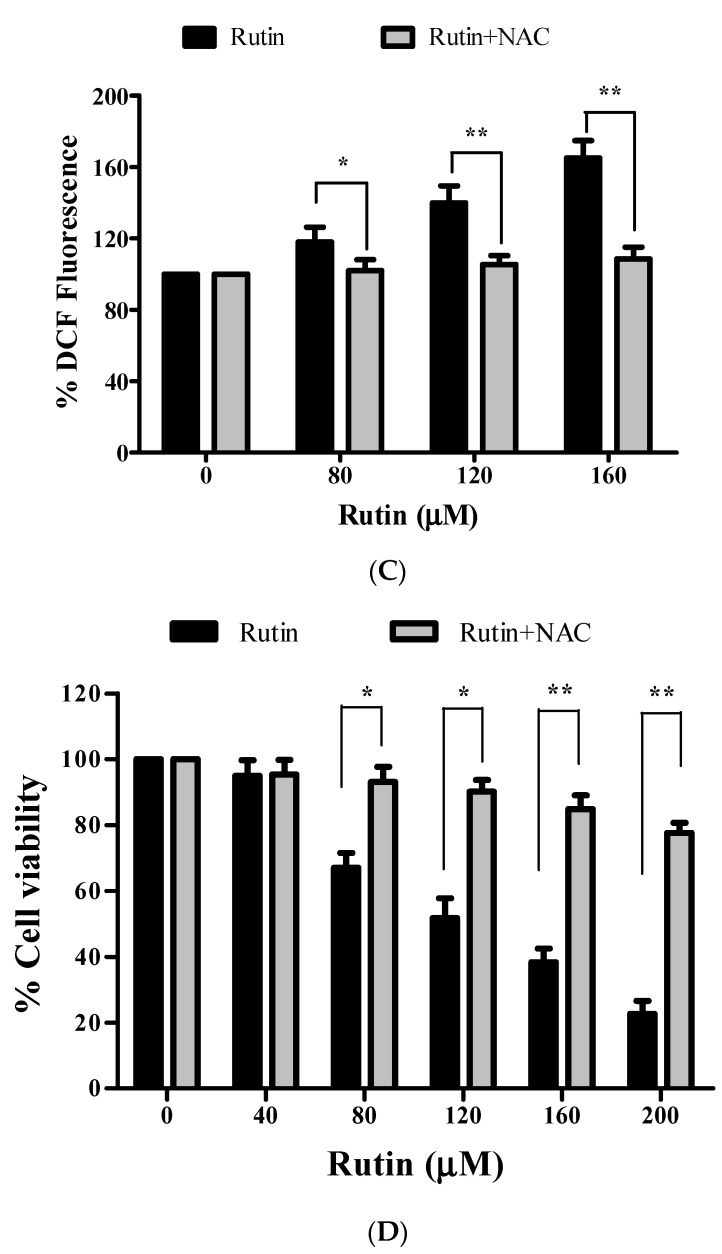
Rutin significantly induces ROS-mediated apoptosis in cervical cancer cells. (**A**) Increased level of ROS generation in rutin-treated SiHa cells stained with by DCFDA staining and examined by fluorescence microscopy. (**B**) Quantification of enhanced percentage of DCFDA fluorescence in rutin-treated SiHa cells. (**C**) Comparative analysis of ROS generation in SiHa cells pretreated with a NAC (ROS inhibitor) and then with different concentrations of rutin. (**D**) Percent cell viability of SiHa cells pretreated with NAC and then with rutin for 24 h assessed by MTT assay. The results are represented as mean ± SEM of three independent experiments (* *p* < 0.01, ** *p* < 0.001 compared with control group).

In order to find out whether rutin mediates its apoptotic effects through ROS generation, intracellular levels of ROS were analyzed by employing fluorescence microscopy (CM-H2DCFDA fluorescent probe). Figure 6A clearly shows an increased intracellular ROS level (significant fluorescence intensity) in rutin-treated cells for 12 h. Quantitative analysis also presented augmented ROS production in a dose-dependent manner (Figure 6B). Further, to corroborate the rutin-mediated augmentation of ROS level, SiHa cancer cells were pretreated with ROS inhibitor (NAC, *N*-acetyl-*L*-cysteine). Quantitative analysis displayed the attenuation of elevated ROS level in NAC (10 mm) pretreated SiHa cancer cells, which strongly substantiated our research that rutin could enhance the ROS level in SiHa cancer cells (Figure 6C). To establish the ROS involvement in rutin-induced cytotoxicity in SiHa cancer cells, we investigated their effects in NAC (10 mm) pretreated SiHa cancer cells by MTT assay. Pretreated SiHa cells exhibited a significant reduction in cytotoxicity caused by rutin (Figure 6D). Hence, these findings indicated that augmented ROS generation is crucial for rutin-induced apoptosis in SiHa cancer cells.

## 4. Discussion

To date, numerous reports have demonstrated the crosstalk between *Jab1* and oncogenic signaling pathways, thereby suggesting it as a potential therapeutic target in several carcinomas including cervical cancer. Therefore, we focused our study toward exploring the inhibitory potential of rutin against *Jab1* in SiHa cervical cancer cells as well as the series of events associated with apoptosis induction that have not been explored till now. Our research findings proved our hypothesis via establishing strong inhibitory efficacy of rutin against *Jab1* in SiHa cancer cells. Deregulated apoptosis has been associated with several human malignancies including cancer [24,25]. *Jab1* has been crucially involved in regulating a series of tumor biological processes such as apoptosis, invasion, cellular proliferation, migration, and cell cycle [26,27,28,29]. Several types of research have thus been emphasizing exploiting the potential of phytocompounds for targeting these aberrant biomarkers via apoptosis induction in developing a better therapeutic approach against cancer [30,31,32,33,34]. One such phytochemical, rutin (glycosylated polyphenolic phytocompound), has been reported in various fruits and vegetables and has exhibited significant anticancer potential in various carcinomas such as bladder, cervical, liver, stomach, prostate, and lung cancers. Our recently published research has also supported the anticancerous efficacy of rutin in cervical cancer, which has further promoted us to unravel the inhibitory potential of rutin against a potent multifunctional oncogene that has been crucially involved in the progression of various cancers [35,36,37].

Firstly, we employed MTT and LDH assays to establish the cytotoxic potential of rutin in SiHa cancer cells and our results exhibited significant growth inhibitory effect in a dose-dependent manner (Figure 1A,B). However, rutin does not cause any significant cytotoxicity in normal cells as reported in our previous study [36]. RT-PCR assay was then performed to establish the inverse association between rutin and *Jab1* expression in cervical cancer that has not been explored yet. Our findings revealed that rutin treatment resulted in a significant reduction in the mRNA expression of *Jab1* (Figure 2A). This further prompted us to elucidate the molecular mechanisms of *Jab1*-induced apoptotic signaling pathways that regulate the biological behaviors of cervical cancer cells.

Several reports have explained the association of *Jab1*/*p27* inverse correlation with the phytocompound-mediated apoptosis induction in cancer cells [37,38,39,40]. *Jab1* executes its biological role by promoting the degradation of several tumor suppressor genes including *p27* and *p53* via translocating them from the nucleus to cytoplasm [41,42]. Sang et al. have also supported the fact that *Jab1* inhibition promoted apoptosis induction via *p53*-mediated apoptotic pathways in gastric carcinoma [43]. Therefore, we investigated the effect of rutin treatment on mRNA expression of *p53* and *p27* in SiHa cells. The qRT-PCR results showed significant upregulation of *p27* and *p53* levels in rutin-treated SiHa cells in a dose-responsive manner (Figure 2B).

It was further reported that *Jab1* knockdown resulted in modulation of *Bax* (upregulation) and *Bcl-2* (downregulation) expression, which subsequently led to cancer cell growth inhibition through apoptosis induction [44]. It is a well-known fact that *Bax* (pro-apoptotic protein) plays a critical role in the regulation of the intrinsic apoptotic pathway through pore formation in the outer mitochondrial membranes, thereby resulting in the cytochrome c leakage, which is inhibited by *Bcl-2* (anti-apoptotic protein) [45]. In the present study, rutin treatment resulted in increased *Bax* mRNA level and reduced *Bcl-2* mRNA expression levels in cervical cancer SiHa cells (Figure 2C). These results projected the modulation of apoptosis-related genes as one of the molecular mechanisms associated with apoptosis induction in rutin-treated SiHa cells.

The hallmarks of apoptosis are based on morphological characteristics such as nuclear and chromatin condensation, which are further associated with rounding up of the cell, cellular volume reduction, and apoptotic body formation [46,47,48,49]. The DAPI staining method was, therefore, utilized to analyze the nuclear morphology in rutin-treated SiHa cells, and the obtained results revealed the increase in nuclear condensation in a dose-dependent manner (Figure 3A). Additionally, the Annexin V- FITC/PI staining method was performed to quantify the early and late apoptotic cells. The results of the Annexin V FITC/PI assay show a dose-responsive increase in the number of apoptotic cells after rutin treatment at 24 h (Figure 3B,C).

Numerous studies have reported the implication of the mitochondrial apoptotic pathway in the apoptosis induction potential of phytocompounds in several cancers [50,51,52]. MitoTracker and Rhodamine dye analysis displayed depolarized MMP (mitochondrial membrane potential) of rutin-treated SiHa cells (Figure 4A,B). Moreover, rutin treatment also enhances cytochrome c release into the cytosol, which is needed for subsequent activation of *Caspase-3* and -9 (Figure 4C). All apoptotic signaling pathways have been reported to be activated by a cascade of caspases (family of cysteine proteases) that cleave proteins at aspartate residues [50,51]. The results of the present study revealed that rutin treatment led to a significantly stimulated caspase activity and mRNA expression level of both the caspases in cervical cancer cells (Figure 5A–D). These data indicated that rutin induces apoptosis in cervical cancer cells through a caspase-dependent pathway.

Recent research has emphasized oxidative-stress-mediated apoptosis induction as one of the promising therapeutic strategies against cancer cells (not in normal cells) [52]. Elevated ROS levels have been reported to act as prominent intracellular signaling molecules in apoptosis induction [52]. Several phytocompounds have been reported to be ROS inducers in various cancer cells, and in accordance with these findings, rutin treatment also resulted in dose-dependent ROS generation in SiHa cancer cells, which plays an important role in cell apoptosis. Moreover, attenuation of ROS by NAC (*N*-acetylcysteine) significantly inhibited rutin-induced cell death in cervical cancer cells (Figure 6A–D). Taken together, the present study manifested that rutin induces mitochondrial-mediated apoptotic cell death via *Jab1* downregulation and upregulation of tumor suppressor genes in cervical SiHa cancer cells. Altogether, we conclude that rutin may be used as a future anti-cervical cancer agent after in vivo and clinical studies.

## 5. Conclusions

In conclusion, our research highlights that rutin possesses significant growth inhibitory effects on SiHa cervical cancer cells. This is the first study that established a direct association between rutin-mediated *Jab1* downregulation and the growth inhibitory potential of rutin in SiHa cancer cells. Further, we employed several assays to establish a possible mechanism associated with this *Jab1* inhibitory potential of rutin. Altogether, rutin induces apoptosis in cervical cancer cells through *Jab1* downregulation and caspase- and ROS-dependent mitochondrial pathways. Hence, our findings suggest that rutin could be a promising therapeutic agent for better management of cervical cancer with minimal side effects and toxicity against normal cells. Further research is still warranted to elucidate the anticancer potential of rutin in vivo.

## Figures and Tables

**Table 1:** Primers used in the study.

Gene	Forward Primer	Reverse Primer
*Bax*	AGGGTGGCTGGGAAGGC	TGAGCGAGGCGGTGAGG
*Bcl-2*	ATCGCTCTGTGGATGACTGAGTAC	AGAGACAGCCAGGAGAAATCAAAC
*Jab1*	GGCGCCTTTAGGACATACC	CATGAAACTCCCTCGTCCC
*Caspase-3*	ACCAAAGATCATACATGGAAGCGA	CGAGATGTCATTCCAGTGCT
*Caspase-9*	TGGTGATGTCGGTGCTCTTG	ACCATGAAATGCAGCGAGGA
*p27*	TCTGGAACAGCGTGCCATTGATCT	ATTACTGAGGGCCACTTCCACCTT
*p53*	ACTAAGCGAGCACTGCCCAA	ATGGCGGGAGGTAGACTGAC
*GAPDH*	AAGTTCAACGGCACAGTCAAGG	CATACTCAGCACCAGCATCACC

## Data Availability

All data are available from the corresponding author on reasonable request.
